# Genomic characterization of a multidrug-resistant uropathogenic *Escherichia coli* and evaluation of *Echeveria* plant extracts as antibacterials

**DOI:** 10.3934/microbiol.2024003

**Published:** 2024-01-17

**Authors:** Ana M. Castañeda-Meléndrez, José A. Magaña-Lizárraga, Marcela Martínez-Valenzuela, Aldo F. Clemente-Soto, Patricia C. García-Cervantes, Francisco Delgado-Vargas, Rodolfo Bernal-Reynaga

**Affiliations:** Unidad de Investigaciones en Salud Pública “Dra. Kaethe Willms”, Facultad de Ciencias Químico-Biológicas. Universidad Autónoma de Sinaloa. Ciudad Universitaria, Culiacán, Sinaloa, México

**Keywords:** Uropathogenic *Escherichia coli*, multidrug resistance, genome sequencing, *Echeveria* extract, antibacterial effect, bacterial adherence

## Abstract

Uropathogenic *Escherichia coli* (UPEC) is the most common bacterial agent associated with urinary tract infections, threatening public health systems with elevated medical costs and high morbidity rates. The successful establishment of the infection is associated with virulence factors encoded in its genome, in addition to antibacterial resistance genes, which could limit the treatment and resolution of the infection. In this sense, plant extracts from the genus *Echeveria* have traditionally been used to treat diverse infectious diseases. However, little is known about the effects of these extracts on bacteria and their potential mechanisms of action. This study aims to sequence a multidrug-resistant UPEC isolate (UTI-U7) and assess the multilocus sequence typing (MLST), virulence factors, antimicrobial resistance profile, genes, serotype, and plasmid content. Antimicrobial susceptibility profiling was performed using the Kirby-Bauer disk diffusion. The antibacterial and anti-adherent effects of the methanol extracts (ME) of *Echeveria* (*E. craigiana*, *E. kimnachii*, and *E. subrigida*) against UTI-U7 were determined. The isolate was characterized as an O25:H4-B2-ST2279-CH40 subclone and had resistant determinants to aminoglycosides, β-lactams, fluoroquinolones/quinolones, amphenicols, and tetracyclines, which matched with the antimicrobial resistance profile. The virulence genes identified encode adherence factors, iron uptake, protectins/serum resistance, and toxins. Identified plasmids belonged to the IncF group (IncFIA, IncFIB, and IncFII), alongside several prophage-like elements. After an extensive genome analysis that confirmed the pathogenic status of UTI-U7 isolate, *Echeveria* extracts were tested to determine their antibacterial effects; as an extract, *E. subrigida* (MIC, 5 mg/mL) displayed the best inhibitory effect. However, the adherence between UTI-U7 and HeLa cells was unaffected by the ME of the *E. subrigida* extract.

## 1. Introduction

Urinary tract infections (UTIs) are among the most common bacterial infections that encompass a wide variety of clinical features and represent a primary public health problem. UTIs are estimated to affect 150 million people worldwide annually, causing high medical costs and morbidities, that can ultimately lead to a significant mortality rate [1]–[3]. Uropathogenic *Escherichia coli* (UPEC) is the most common causative agent of UTIs, accounting for 70–95% of community-acquired UTIs and 50% of nosocomial UTIs. The human intestine functions as a reservoir for UPEC, and it is believed to disseminate to the urinary tract from the host's intestinal microbiota. Subsequently, UPEC colonizes the periurethral area, ascends to the bladder, and advances through the ureters to the kidneys [4]–[6]. This pathogenic mechanism involves different steps, which includes adherence, colonization, ascension, biofilm formation, and invasion. Thus, UPEC strains have acquired virulence factors (VFs) through horizontal gene transfer that allows them to adapt to new niches and potentially cause damage to the host. The VFs are adhesion factors (for example P, type 1, S fimbriae), toxins (e.g., α-hemolysin, necrotizing cytotoxic factor (CNF1)), and various siderophores (e.g., IroN, IutA, FyuA) [7]–[11]. Besides this, the dissemination of antibiotic resistance genes results in ineffective UTI treatment and the emergence of multidrug-resistant (MDR) pathogens related to the inappropriate use of antibiotics that are prescribed without being supported by bacterial susceptibility testing [12]. These MDR strains have become a clinical problem in recent decades, particularly in patients with recurrent UTIs, owing to limited treatment options. Both pathogenesis and antimicrobial resistance synergize to avoid bacterial eradication and the establishment of persistent infections; therefore, the search for new therapeutic alternatives is constant [13]. Traditionally, plant-based medicines have been used to treat diseases associated with microbial infections. Bioactive compounds found in plants, such as alkaloids, phenols, saponins, and flavonoids, have shown antimicrobial activity relevant for the prevention and treatment of infectious diseases, including UTIs [13],[14]. A current proposal to counteract these infections are plants belonging to the Crassulaceae family, mainly plants from the genus *Echeveria* [15]. These plants are used to treat diseases and symptoms, such as diarrhea, oral herpes, inflammation, stomach aches, and fever. Previous evidence from *Echeveria gibbiflora* extracts have shown contraceptive effects [16]; additionally, extracts from *Echeveria leucotricha*, *E. kimnachii, E. craigiana*, and *E. subrigida* have shown antimicrobial activity against Gram-negative and Gram-positive bacteria that have been associated with metabolites such as α-tocopherol and phenolics [17],[18]. Recently, it has been shown that *Echeveria* methanol extracts have bacteriostatic effects on diarrheagenic strains and might be useful as an antibacterial agent to treat infections [19]. However, there is no evidence regarding *Echeveria* extracts as a treatment for UTIs or UPEC isolates. Here, we describe a MDR UPEC isolate genome from a patient in Sinaloa, Mexico, and how an *Echeveria* plant extract could be a potential treatment for these pathogenic strains.

## 2. Materials and methods

### 2.1. Bacterial isolation and identification

*Escherichia coli* isolate UTI-U7 was isolated from a pregnant patient of the Gynecology and Obstetrics services at the Civil Hospital in Culiacan, Sinaloa, Mexico. All procedures were approved by the Ethics Committee of the “Centro de Investigación y Docencia en Ciencias de la Salud de la Universidad Autónoma de Sinaloa” (registration CONBIOETICA-25-CEI-001-20180523). Samples from the first morning micturition were collected in a sterile container by a mid-stream catch, inoculated in cystine–lactose–electrolyte-deficient (CLED) (MCDLab, Mexico) and MacConkey (MCDLab, Mexico) agar plates, and incubated at 37 °C for 18–24 hours for the macroscopic identification of colonies. An asymptomatic urinary infection was diagnosed after urine examination and urine culture. The identity of the UTI-U7 isolate was confirmed using conventional biochemical tests. The UTI-U7 isolate was stored in a Brain Heart Infusion (BHI, Difco, MA, USA) broth with 8% dimethyl sulfoxide (DMSO) at −80 °C until use.

### 2.2. Antimicrobial susceptibility testing (AST)

The antimicrobial resistance profile of the UTI-U7 isolate was determined using the Kirby-Bauer disk diffusion method following the Clinical and Laboratory Standard Institute guidelines (CLSI) [20],[21]. First, a tube with 4 mL of Mueller Hinton (MH) culture broth (MCDLab, Mexico) was inoculated with the U7 isolate and adjusted to 1 × 10^8^ CFU/mL. Then, the previously prepared inoculum was streaked onto MH agar plates. Finally, antibiotic disks were placed on the agar surface. The antibiotic disk (Diagnostic Research, ID, Mexico) contained amikacin (AK, 30 µg), ampicillin (AM, 10 µg), carbenicillin (CB, 100 µg), cephalothin (CF, 30 µg), cefotaxime (CFX, 30 µg), ciprofloxacin (CPF, 5 µg), chloramphenicol (CL, 30 µg), gentamicin (GE, 10 µg), netilmicin (NET, 30 µg), nitrofurantoin (NF, 300 µg), norfloxacin (NOF, 10 µg), and sulfamethoxazole/trimethoprim (STX, 25 µg). Plates were incubated at 37 °C for 16–18 h. Following incubation, the inhibition zones were measured and interpreted according to the CLSI criteria (M100 ED32:2022). *E. coli* ATCC 25922 was used as a quality control for this assay.

### 2.3. DNA extraction and Whole-Genome Sequencing (WGS)

DNA extraction and sequencing was performed as previously described [22]. A single *E. coli* colony was grown in Luria-Bertani (LB) broth overnight at 37 ± 2 °C under aerobic conditions. Genomic DNA (gDNA) was extracted using the Wizard Genomics DNA Purification Kit (Promega Corporation, Madison, WI, USA) following the manufacturer's instructions. The quality of gDNA (i.e., purity and integrity) was assessed by a spectrophotometry method using a DeNovix DS-11 spectrophotometer (DeNovix Inc., Wilmington, DE, USA) and 1.0% agarose gel electrophoresis, respectively. Subsequently, the gDNA was quantified using a Qubit 2.0 fluorometer (Invitrogen, Carlsbad, CA, USA) and appropriately adjusted for paired-end (PE) DNA library preparation using the Nextera XT DNA Library Preparation kit (Illumina, San Diego, CA, USA) following the manufacturer's protocol. Genomic sequencing was performed on an Illumina MiniSeq instrument using a 2 × 150 bp strategy. The raw genomic data of the UTI-U7 isolate was deposited into the Sequence Read Archive (SRA) (accession number SRR21677288) of the National Center for Biotechnology Information (NCBI) linked to BioProject PRJNA883009.

### 2.4. Read Preprocessing, genome assembly and annotation

A quality control assessment, filtering, and further assembly of raw sequencing data were performed as previously reported [22]. The FastQC program, version 0.11.9 (https://www.bioinformatics.babraham.ac.uk/projects/fastqc/), and Cutadapt, version 2.4 [23], were employed to evaluate and process the raw genomic reads, respectively. For genome assembly and assembly quality assessment, the de novo assembly algorithm by SPAdes, version 3.15.1 [24], and QUAST, version 5.0.2 [25], were used, respectively. Before genome scaffolding, the contigs were analyzed using the KmerFinder web tool, version 3.0.2 [26],[27], from the Center for Genomic Epidemiology (CGE; http://www.genomicepidemiology.org/) and used to obtain a reference genome. UTI-U7 genome scaffolding was performed using the MeDuSa [28] web server (http://combo.dbe.unifi.it/medusa/), chromosomal sequences of *E. coli* strain p4A (RefSeq NZ_CP049085.2), E302 (RefSeq NZ_AP022362.1), SCU-172 (RefSeq NZ_CP054353.1), and an extra chromosomal element (RefSeq NZ_CP015088.1). The Mauve Contig Mover tool of the Mauve version snapshot_2015_02_13 [29],[30] was used to reorder the genomic scaffolds of UTI-U7 against the complete genome of *E. coli* p4A. Scaffolds shorter than 500 bp in length were excluded and annotated by Rapid Annotations using Subsystems Technology (RAST) [31].

### 2.5. In Silico genome characterization

To determine the key epidemiological features of UTI-U7, the assembled genome was subjected to *in silico* phylotyping, *fumC fimH* (CH) typing, serotyping, and multilocus sequence typing (MLST) based on the Achtman seven-loci scheme [32]. Hence, diverse web tools and associated databases were surveyed under default parameter settings unless otherwise specified. The phylogroup assignment was assessed using ClermonTyping, version 21.03 [33] (http://clermontyping.iame-research.center/index.php; accessed on September 17, 2022), which is based on the phylogrouping method developed by Clermont et al. [34]. For CH typing (a two-locus, sequenced-based typing scheme for rapid sequence type determination), CHTyper, version 1.0 [35], was used to determine the allelic variants of *fumC* and *fimH* genes, and subsequently define the clonotype CH (*fumC*/*fimH*) [36]. The serotype was predicted using SerotypeFinder, version 2.0.1 [37]. MLST was performed using MLST, version 2.0.9 [38], selecting the “*Escherichia coli* #1” scheme option.

Moreover, the presence of antimicrobial resistance genes (ARGs), virulence-associated genes (VAGs), and plasmids in the UTI-U7 genome were analyzed. The acquired ARGs and chromosomal mutations involved in antibiotic resistance were detected using ResFinder, version 4.1 [39],[40]. VAGs were identified using VirulenceFinder, version 2.0.3 [41],[42]. Plasmids were identified by replicon sequence detection using PlasmidFinder, version 2.0.1, and subtyped with *in silico* plasmid multiLocus sequence typing (pMLST), version 2.0 [43]. To recognize small plasmids, the percent identity (%ID) and coverage thresholds of the PlasmidFinder tool were set to 80% and 60%, respectively, as recommended by Carattoli and Hasman [44]. All of the aforementioned tools are available at the CGE website (http://www.genomicepidemiology.org/ (accessed on September 18, 2022)).

To identify and annotate the prophage regions harbored in the genomic sequence of UTI-U7, the Phage Search Tool Enhanced Release (PHASTER; https://phaster.ca/) server was employed [45] (accessed on September 30, 2022). The PHASTER tool predicts and classifies a prophage region as either intact (score > 90), questionable (score 70–90), or incomplete (score < 70) based on their similarity to known phages, sizes, and phage-related gene content. The protein-coding sequences (CDS) annotated by PHASTER as either “hypothetical protein” from viral origin or “hypothetical” from a bacterial origin underwent BLASTp searches against the non-redundant protein sequences (nr) database (updated version October 30, 2022) using an E-value of 1e^−05^.

### 2.6. Phylogenetic analysis

The phylogenetic analysis of the UTI-U7 isolate was performed using a collection of 42 additional genomes belonging to the ST2279 lineage retrieved from EnteroBase (https://enterobase.warwick.ac.uk/species/index/ecoli; accessed on November 12, 2022) [46]. A minimum spanning tree (MST) was constructed based on the core-genome MLST (cgMLST) V1 + Hierarchical Clustering (HierCC) V1 scheme from EnteroBase using the GrapeTree function and the rapid neighbor-joining (RapidNJ) algorithm [47]. The serotype, clonotype, acquired determinants, and point mutations related to antimicrobial resistance of the 42 genomes were determined using resources from the CGE, as described above. A complete overview of the epidemiological features, ARGs, and point mutations of the ST2279 isolates are provided in Supplementary Table S1. The phylogenetic tree was visualized and annotated using the iTOL platform, version 6.6 [48]. The branch lengths were used to calculate the cgMLST allelic differences between closely related isolates.

### 2.7. Preparation of methanol extracts (ME)

Leaves of three *Echeveria* species (*E. craigiana, E. kimnachii*, and *E. subrigida*) were collected from communities in Sinaloa, Mexico, and transported to the Chemistry of Natural Products Laboratory. The leaves were freeze-dried, milled, passed through a number-40 mesh, and extracted with methanol (1:20 w/v). Methanol was eliminated by rotary evaporation under vacuum, and the residual organic solvent and water were eliminated by freeze-drying; the residue was the methanol extract (ME), which was stored at −80 °C until use [19]. The chemical characterization of the ME of the *Echeveria* species has been previously described [18]. The ME was dissolved in water to obtain a solution with a concentration twice the maximum value to be evaluated. The dilution was sterilized using a 0.45 µm filter, and the filtrate was recovered in a sterile vial. Two-fold serial dilutions of different concentrations were prepared from the sterile extract in a sterile vial using water as the solvent.

### 2.8. Antibacterial activity assay

The minimal inhibitory concentration (MIC) values were determined using the microdilution method as described elsewhere [18]. The UTI-U7 isolate and quality control strain *Escherichia coli* ATCC^®^25922 (ATCC^®^, Manassas,VA) were initially grown on trypticase soy agar (TSA, MCDLab, Mexico) plates for 18-20 h at 37 °C. For inoculum preparation, a few colonies were suspended in 2 mL of a sterile saline solution (0.85% w/v NaCl). The density was adjusted to 1 × 10^8^ CFU/mL (0.5 McFarland) and subsequently diluted to obtain a suspension of 1 × 10^6^ CFU/mL. The MIC assay was performed in sterile 96-well plates. A 50 µL bacterial suspension was added to the wells and mixed with 50 µL of each extract at different concentrations (1000–15,625 µg/mL). Then, the plates were incubated at 37 °C for 18–20 h. The MIC value corresponded to the first well at which no turbidity or growth button was observed. Gentamicin (0.125–16 µg/mL; SIGMA Chemical Company, Missouri, USA) was used as the positive control.

### 2.9. Cell culture

HeLa cells (ATCC^®^ CCL-2, Manassas, VA), which are a cervical adenocarcinoma epithelial cell line, were grown in high-glucose Dulbecco's modified eagle medium (DMEM) (Gibco^®^, USA) supplemented with 10% fetal bovine serum, 100 U/mL penicillin, 100 µg/mL streptomycin, and 0.025 µg/mL amphotericin B (Gibco^®^, USA). Cells were maintained in a 5% CO_2_/95% air atmosphere. For the adherence assay, the cells were seeded onto 24 wells plates and grown for 24 h to obtain a cell monolayer.

### 2.10. Inoculum preparation

An inoculum was prepared as previously described [49]. A well-defined colony was grown overnight (ON) with continuous shaking at 37 °C in LB broth (MCDLab, Mexico). Next, 25 mL of LB broth was inoculated with 1 mL of the ON culture and incubated for 3 h at 37 °C. The bacterial concentration was adjusted to 1 × 10^6^ cells/mL, and then 1 mL of the bacterial inoculum was treated with 1 mL of ME for 3 h at 37 °C. After incubation, treated bacterial cells were centrifuged at 8000 RPM and washed twice with 1 mL of 1x phosphate-buffered saline (PBS). Finally, cells were resuspended in 1 mL of DMEM for adherence assays. Untreated bacterial cells were used as controls.

### 2.11. Adherence assay

Bacterial adherence assays were performed as previously described with some modifications [49],[50]. HeLa cells [50,000 cells/well] were infected with the treated and untreated bacteria at 37 °C for 3 h in a 5% CO_2_ atmosphere. Then, the medium was removed, and the monolayer was washed with 1X PBS. The attached cells were lysed with 1 mL of 1X PBS solution containing 1% Triton X-100 (ThermoFisher Scientific, Waltham, MA, USA) and 0.01% sodium dodecyl sulfate (SDS). The lysed cells with attached bacteria were collected, serially diluted by ten-fold, and plated on TSA plates, which were incubated at 37 °C for 18 to 24 h. Colony-forming-units (CFU) were determined using the standard viable count method. Assays were performed in triplicate.

### 2.12. Statistical analysis

The results are presented in the subsequent figures and tables. CFU count results were analyzed through a t-test using the following analytical software: Stata Intercooled, version 13.1, and GraphPad Prism, v 8.0.1.

## 3. Results

### 3.1. General features of UTI-U7 and genome sequencing

UTI-U7 was recovered in 2019 during a routine urine culture screening of a multiparous 23-year-old pregnant woman with asymptomatic bacteriuria, a history of recurrence, and no active or recent treatment for urinary infection. UTI-U7 was subjected to WGS to elucidate the genetic relatedness and content of the genes involved in antimicrobial resistance, virulence, plasmids, and prophages. Genomic sequencing generated a total of 7,695,494 PE reads, of which 88.7% were preserved after quality trimming, filtering, and assembly into scaffolds, yielding a depth of coverage of 148X. The final assembled genome resulted in 42 scaffolds (> 500 bp) with a genomic length of 5,380,170 bp and a GC content of 50.72%. Gene annotations by RAST predicted 5569 protein-coding sequences (CDS) distributed in 391 subsystems, nine ribosomal RNAs (rRNA), and 84 transfer RNAs (tRNA).

### 3.2. In silico typing of UTI-U7

The serotype, phylotype, sequence type (ST), and clonotype of UTI-U7 were identified through *in silico* characterization. Typing of the somatic (O) and flagellar (H) antigen-encoding genes using the SerotypeFinder tool predicted that UTI-U7 expresses an O25:H4 serotype. The phylogroup assignment determined that it belongs to the B2 phylogroup. The MLST analysis assigned UTI-U7 to ST2279 based on the allelic variant combination of seven housekeeping genes (*adk*-53, *fumC*-40, *gyrB*-47, *icd*-13, *mdh*-36, *purA*-8, and *recA*-29), whereas CH typing assigned UTI-U7 to clonotype CH40-5. Accordingly, the UPEC isolate U7 was characterized as an O25:H4-B2-ST2279-CH40-5 subclone.

### 3.3. Resistance profile, antimicrobial-resistance genes (ARGs) and Virulence-Associated Genes (VAGs) in UTI-U7

The antimicrobial resistance profile of the UTI-U7 isolate was determined by disk diffusion according to CLSI standards. Hence, the U7 isolate can be cataloged as a MDR bacteria with resistance to different classes of antibiotics such as ampicillin, carbenicillin, cephalothin, and cefotaxime, thus suggesting the presence of extended-spectrum β-lactamases (ESBL). Additionally, resistance to ciprofloxacin, norfloxacin, gentamicin, chloramphenicol, and nitrofurantoin was observed (Table 1).

WGS analysis using ResFinder confirmed the presence of ARGs in the UTI-U7 genome. A total of eight determinants conferring resistance to aminoglycosides, β-lactams, fluoroquinolones/quinolones, amphenicols, and tetracyclines antimicrobial classes were detected, of which genes encoding resistance to aminoglycosides accounted for the majority. Four allelic variants encoded two distinct aminoglycoside-modifying enzymes (AMEs): aminoglycoside *N*-acetyltransferases (*aac(3)-Iia*, *aac(6′)-Ib-cr*) and aminoglycoside *O*-phosphotransferases (*aph(3′)-Ib*, *aph(6)-Id*). Of note, AMEs encoded by *aac(6′)-Ib-cr* expressed bifunctional activity, thus conferring reduced fluoroquinolone susceptibility. Two β-lactamase genes were identified in the present study: the *bla_CTX-M-15_* gene encoding the ESBL CTX-M-15 enzyme, and the *bla_OXA-1_* gene encoding a member of class D β-lactamases with reduced susceptibility to penicillin/β-lactamase inhibitor combinations.

Analysis of the chromosomal genes encoding quinolone targets identified point mutations leading to multiple non-synonymous substitutions in GyrA (S83L, D87N), ParC (S80I, E84V), and ParE (I529L). Except for the *aac(6′)-Ib*-cr gene, no other plasmid-mediated quinolone resistance (PMQR) determinants were observed (i.e., *oqxAB*, *qepA*, or *qnr*). UTI-U7 harbored other important determinants involved in amphenicol and tetracycline resistance, such as *the catB3* and *tet(A)* genes. However, phenotypic resistance to tetracycline has not been tested. The *sitABCD* operon was identified, thus contributing to the resistance to disinfectants, such as hydrogen peroxide. Surprisingly, no trimethoprim, sulfonamide, or class 1 integron genes were observed, as neither were carbapenem- or colistin-resistance determinants.

Regarding the virulence potential of UTI-U7, the Virulence Finder tool corroborated the ExPEC status and its UPEC nature by identifying 21 ExPEC-associated virulence genes, some of which were previously detected by PCR in this study (data not shown). These genes were categorized depending on the pathogenic process in which they are involved (adherence, iron uptake, protectin/serum resistance, and toxin production). Thus, the genes *iha*, *hra*, *papA_F19/F20*, *papC*, and *yfcV* were grouped into the adherence category. The genes *chuA*, *fyuA*, *irp2*, *iucC*, and *sitA* were grouped into the iron uptake category, as each is part of a distinct gene cluster encoding iron acquisition systems (i.e., Fe ion transporters and siderophores). In the protectin/serum resistance group, genes involved in capsular polysaccharide biosynthesis (*kpsE*, *kpsMII_K5*) and complement resistance/serum survival (*iss*, *ompT*, *traT*) were identified. Four genes directly involved in synthesis and toxin expression were allocated to the toxin-production category. The *cnf1* gene encodes cytotoxic necrotizing factor 1 (CNF1), while the *hlyA* gene is part of the *hly* operon that encodes α-hemolysin. The *sat* gene encodes a member of the serine protease autotransporter of the *Enterobacteriaceae* (SPATE) family named secreted autotransporter toxin (Sat), and the *usp* gene encodes the uropathogenic specific protein (Usp), which is a bacteriocin-like protein with genotoxic activity. Additionally, the hexosyltransferase homolog (*capU*) and double tellurium ion resistance protein (*terC*) encoding genes were identified. Importantly, no genetic markers specific to DEC pathotypes were identified in the genomic survey.

### 3.4. Plasmid Content and Prophage Regions in UTI-U7

Based on replicon sequence analysis, PlasmidFinder identified five replicons belonging to the Inc group F: IncFIA, IncFIB (AP001918), and three IncFII. When the FAB formula was applied to further subtype the IncF plasmid replicons (pMLST), the putative F plasmid was assigned to ST [F40:A4:B1] as the best-matching hit. Nonetheless, because multiple hits on the IncFII-type replicon surged at different positions and %ID [IncFII/95.42%, IncFII/95.79%, and IncFII(pHN7A8)/98.08%] in the genome, various alleles for the FII replicon were identified (i.e., F31, F36, and F40). Therefore, multiple STs might be assigned. Furthermore, close examination of the scaffolds containing plasmid replicons indicated the presence of a few ARGs and VAGs. In particular, the fragment containing both IncFII–IncFII(pHN7A8) replicons harbored the aminoglycoside resistance genes *aac(3)-IIa*, *aph(6)-Id*, and *aph(3″)-Ib*, whereas the scaffolds with replicons IncFIA and IncFIB(AP001918) harbored *traT* (double copy) and *iha* genes, respectively.

PHASTER identified 16 prophage-like elements in the associated regions. Six of them were predicted as intact, seven as incomplete, and three as questionable (Table 2). Their size ranged from 4.2 kb to 71.1 kb; among the most prevalent identified phages, some showed homology to the *Enterobacteria* phage BP-4795, the *Enterobacteria* phage phiP27, and the *Escherichia* phage DE3. Putative antimicrobial resistance determinants or virulence factors were identified by BLASTing hypothetical CDSs for potential cargo of prophages, regardless of viral (146) or bacterial (104) origin. This examination did not reveal significant results, as a few hypothetical proteins were related to antimicrobial resistance or virulence properties. Instead, most maintained a hypothetical/uncharacterized protein status, whereas others only contained a conserved domain of unknown function (DUF).

**Table 1.** General features observed after genome sequencing of UPEC U7 isolate.

Antibiotic resistance profile^a^	Antimicrobial resistance genes						Additional resistance genes	Virulence genes
	β-lactams	Aminoglycosides	Fluroquinolones/quinolones	Amphenicols	Tetracyclines	QRDR mutations		
AM, CB, CP, CFX, CPF, GE, NOF	*bla_CTX-M-15_* *bla_OXA-1_*	*aac(3)-Iia* *aac(6′)-Ib-cr* *aph(3″)-Ib* *aph(6)-Id*	*aac(6′)-Ib-cr*	*catB3*	*tet(A)*	*gyrA-S83L, D87N* *parC-S80I, E84V* *parE I529L*	*sitABCD*	Adherence: *iha*, *hra*, *papA_F19/F20*, *papC*, *yfcV* Iron uptake: *chuA*, *fyuA*, *irp2*, *iucC*, *sitA* Serum resistance: *kpsE*, *kpsMII_K5, iss*, *ompT*, *traT* Toxins: *cnf1, hlyA, sat, usp* Other genes: *capU, terC*

^a^Resistance profile by Kirby-Bauer method: AM: Ampicilin, CB: Carbenicilin, CP: Cephalotin, CFX: Cefotaxime, CPF: Ciprofloxacin, GE: Gentamicin, NOF: Norfloxacin.

**Table 2.** Prediction of putative prophage regions in the UTI-U7 isolate.

Region	Length (kb)	Completeness	Score	CDS	Phage (Hit genes counts) ^a^	GC %	RefSeq Accession
1	8.3	Incomplete	40	9	*Enterobacteria* phage BP-4795 (2)	50.3	NC_004813.1
2	5.4	Incomplete	60	9	*Bacillus* phage BalMu-1 (1)	54.4	NC_030945.1
3	53.8	Intact	150	64	*Enterobacteria* phage mEp460 (35)	50.3	NC_019716.1
4	15	Intact	110	19	*Escherichia* phage 500465-1 (4)	53	NC_049342.1
5	67.9	Intact	150	61	*Enterobacteria* phage SfV (22)	50.4	NC_003444.1
6	43.4	Intact	150	60	*Pectobacterium* phage ZF40 (12)	48	NC_019522.1
7	13.4	Incomplete	30	20	*Enterobacteria* phage phiP27 (7)	48	NC_003356.1
8	16.2	Incomplete	30	22	*Escherichia* phage DE3 (14)	54.4	NC_042057.1
9	28.3	Intact	150	34	*Enterobacteria* phage BP-4795 (21)	57.2	NC_004813.1
10	25.2	Incomplete	20	33	*Enterobacteria* phage phiP27 (9)	47.9	NC_003356.1
11	44.9	Questionable	90	54	*Escherichia* phage DE3 (22)	51.2	NC_042057.1
12	36.8	Questionable	90	50	*Burkholderia* phage BcepMu (28)	54.9	NC_005882.1
13	71.1	Intact	150	79	*Escherichia* phage pro483 (30)	51.4	NC_028943.1
14	19.2	Incomplete	30	18	*Escherichia* phage P88 (9)	52.5	NC_026014.1
15	4.2	Questionable	70	7	Stx2-converting phage Stx2a_F451 (3)	54	NC_049924.1
16	5.1	Incomplete	60	8	Stx2-converting phage Stx2a_WGPS9 (2)	55.7	NC_049923.1

^a^ Phage with the highest number of CDS similar with those in the region.

Notably, some hypothetical proteins incorrectly annotated by PHASTER were proteins involved in both bacterial and viral metabolic, regulatory, or transport functions. Among the antimicrobial resistance determinants, two proteins, which are members of the small multidrug resistance (SMR) and major facilitator superfamily (MFS) transporter superfamilies, were recognized in *Enterobacteria* phage BP-4795 (region 1) and *Escherichia* phage pro483 (region 13), respectively. However, the former is a truncated protein because a nonsense mutation was introduced into the DNA sequence. Besides the Iss/Bor protein, which is involved in bacterial serum resistance, an Ig-like domain-containing protein showing homology to a putative adhesin was noted, both of which were harbored in the *Escherichia* phage DE3 (region 11). Likewise, a TonB-dependent receptor, which is a member of the outer membrane receptor family that mediates substrate-specific transport, including siderophores, was recognized in *Escherichia* phage 500465-1 (region 4).

### 3.5. Phylogenetic analysis

**Figure 1.**
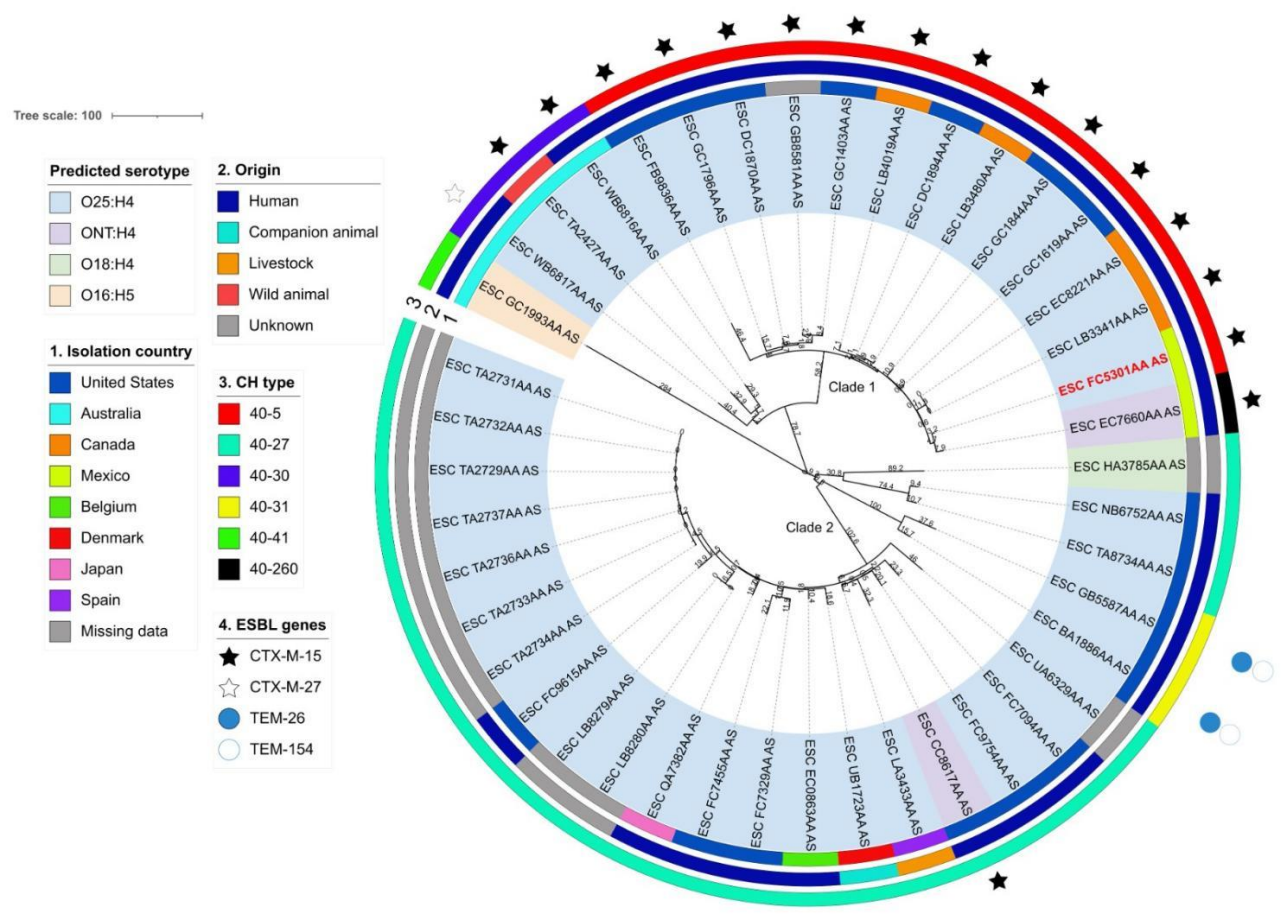
Core genome MLST-based phylogenetic tree of the *E. coli* ST2279 lineage. The phylogeny was constructed using the rapid neighbor-joining (RapidNJ) algorithm and the core-genome MLST (cgMLST) V1 + Hierarchical Clustering (HierCC) V1 scheme from EnteroBase. The nodes were named according to the isolate ID designations from EnteroBase. UTI-U7 isolate (ESC_FC5301AA_AS) is highlighted in red. The scale bar indicates the number of allele differences.

According to an EnteroBase search (accessed on November 12, 2022), 43 *E. coli* isolates constitute the ST2279 lineage worldwide, most of which are from the United States (17), Australia (4), and Canada (4); however, a significant number of isolates (11) lack geographical information. Overall, the global collection of the ST2279 lineage is predominantly comprised of human-derived isolates associated with urinary tract and bloodstream infections and, to a lesser extent, isolates from animal hosts. The phylogenetic reconstruction showed that the ST2279 lineage is clustered into two major clades (1 and 2) and is mainly restricted by the serotype (O25/ONT:H4) and clonotype CH 40-5 and CH 40-27, respectively (Figure 1). Clade 1 housed *E. coli* genomes collected from North American countries, whereas clade 2 clustered genomes originated from diverse geographical locations.

UTI-U7 (isolate ID ESC_FC5301AA_SS) was grouped in clade 1; according to the cgMLST analysis, it was most closely related to the isolate ESC_EC7660AA_AS, which is another Mexican *E. coli* strain isolated during the same year and geographical location, differing by only 11 cgMLST alleles (Figure 1). The *in silico* characterization of antimicrobial resistance corroborated that all *E. coli* of lineage ST2279 studied were multidrug-resistant strains with varying ARGs cargoes (Table S1). Generally, all *E. coli* strains encode at least one determinant related to aminoglycosides, β-lactams, tetracyclines, trimethoprim, or sulfonamide-acquired resistance, as well as an amino acid substitution in the quinolone-drug targets (GyrA, ParC, or ParE).

Further analysis of the ARGs content from clade 1 strains revealed a key feature: all exhibited multiple non-synonymous mutations in the quinolone resistance-determining region (QRDR), the PMQR *aac(6′)-Ib-cr* gene, and two distinct β-lactamase genes (*bla_OXA-1_* and *bla_CTX-M-15_*). Conversely, none of the clade 2 strains except for one (ESC_CC8617AA_AS) possessed the *aac(6′)-Ib-cr* gene, an ESBL- or OXA-type β-lactamase variant, or multiple mutations in the QRDR. Instead, the majority were characterized as harboring a single mutation in *ParE* (I529L) and the ARGs variants conferring resistance to aminoglycosides (*aph(3″)-Ib*, *aph(6)-Id*), β-lactams (*bla_TEM-1B_*), sulfonamides (*sul2*), tetracyclines (*tet(A)*), and trimethoprim (*dfrA8*). Notably, the two clonotype CH 40-31 strains (ESC_BA1886AA_AS and ESC_GB5587AA_AS) shared a similar ARGs profile characterized by the co-harboring of multiple TEM-type β-lactamases (TEM-1A, TEM-26, TEM-154, and TEM-189), of which TEM-26 and TEM-154 were ESBL variants, in addition to OXA-2 β-lactamase.

### 3.6. Antibacterial and antiadherent activity of Echeveria extracts on UTI-U7 isolate

In the search for new alternative treatments to combat antimicrobial resistance, the antibacterial effects of three MEs of *Echeveria* species were studied against the UTI-U7 pathogenic isolate. *E. subrigida* showed the lowest MIC against UTI-U7 (5 mg/mL). *E. craigiana* and *E. kimnachi* showed higher MIC values (25 and 50 mg/mL, respectively; Table 3). Based on these observations, and the previous evidence of the presence of secondary metabolites related to its antibacterial activity against Gram-negative bacteria [18],[19], subsequent experiments were performed using the *E. subrigida* extracts.

**Figure 2.**
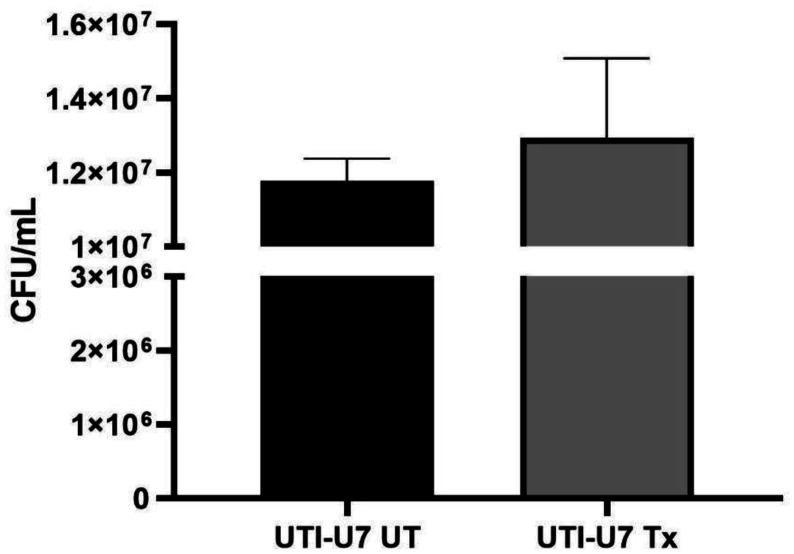
Effect of *Echeveria subrigida* extract on UTI-U7 adherence. Bacterial cells of UPEC isolate were treated with *Echeveria subrigida* methanol extract for 3 h and adherence to HeLa cells was quantified. Colony-forming-units (CFU) were obtained after plating out ten-fold serial dilutions of adhered untreated (UT) and treated (Tx) bacteria. Mean of three independent experiments is shown.

Since bacterial adhesion to epithelial cells is a critical step in the pathogenesis of UPEC, we investigated whether treating UTI-U7 with *Echeveria subrigida* may prevent this isolate from adhering to HeLa cells. The CFU quantification showed an adhesion mean of 1.29 × 10^7^ for treated bacteria and 1.18 × 10^7^ for the untreated bacteria, with no statistical difference among them (*p* = 0.4163; Figure 2). Hence, the extract of *Echeveria subrigida* did not affect UTI-U7 adherence.

**Table 3.** Minimum inhibitory concentration (MIC; mg/mL) of the bacterial growth of UTI-U7 isolate by methanolic extracts of *Echeveria* spp.

Strain	*E. subrigida*	*E. kimnachii*	*E. craigiana*
UTI-U7	5	50	25
*Escherichia coli* ATCC 25922	5	50	25

## 4. Discussion

The primary cause of urinary tract infections is UPEC. These strains contain various virulent factors that enable them to cause infection. Additionally, the likelihood of severe diseases such as septicemia and bacteremia is amplified due to antimicrobial resistance, thus resulting in higher mortality rates. The present study describes a multidrug-resistant UPEC isolated from an asymptomatic patient; this isolate could represent a risk to public health based on its genome analysis, through which we could know the virulence, resistance, and other characteristics that the isolate carries. In this sense, the UTI-U7 isolate belongs to the O25:H4 serotype, which is commonly associated with highly pathogenic strains and is a major producer of hospital and community UTIs [51],[52]. UTI-U7 was assigned to the B2 phylogroup, which is associated with extra-intestinal pathogenic strains and the predominant group in UPEC isolates [33],[53]–[56]. The isolate belongs to the ST2279 lineage (owing to the combination of *adk*-53, *fumC*-40, *gyrB*-47, *icd*-13, *mdh*-36, *purA*-8, and *recA*-29), which is closely related to the ST131 high-risk clone. It has been reported that the only difference between these two groups is a single nucleotide at position 263 of the *purA* gene [57]. Few studies have associated ST2279 with isolates from human samples, such as tracheal secretions, urine, and blood [58],[59], in accordance with the phylogenetic analysis performed in the present study. Additionally, the phylogenetic analysis showed that UTI-U7 is clustered into two clades that contain the genomes of strains isolated in North America (clade 1) and diverse locations around the world (clade 2). These North American isolates included one from Mexico that was isolated in the same year and location, which could be relevant to epidemiological surveillance and of concern to the public health of this country. On the other hand, the phenotypic resistance profile and WGS showed that UTI-U7 has ARGs which can be considered as a MDR isolate with resistance to antibiotics such as aminoglycosides, fluoroquinolones/quinolones, amphenicols, tetracyclines, and β-lactams. The latter was confirmed by identifying the genes for *bla_CTX-M-15_* and *bla_OXA-1_*. In this sense, UPEC strains have been extensively associated with the production of ESBL, especially the CTX-M group, whose presence is associated with co-resistance to aminoglycosides and fluoroquinolones [61], as it was found in UTI-U7. *bla_CTX-M-15_* has been reported as the most prevalent ARG in UPEC strains [60]–[64]. In addition to the *bla_CTX-M-15_* and *bla_OXA-1_* variants, no other determinants encoding beta-lactamase was found in the genomic sequence of the UTI-U7 isolate. Recently, from a genomic perspective, *E. coli* strains have been derived from diverse isolation sources (BioProject PRJNA715781, PRJNA414631, and PRJNA355006) [22]. However, we have not found evidence of the carriage of metallo-beta-lactamases, even when high-risk MDR clones such as ST131, ST410, and ST617 have been noticed. Therefore, extensive epidemiological and genomic surveys are required. On the other hand, the association between *bla_CTX-M-15_* and *bla_OXA-1_* has been previously demonstrated in plasmids of the IncF group, thus reducing the effect of the combination of β-lactam/β-lactamase inhibitors [65]. The UTI-U7 genomic analysis showed the presence of a group of plasmid replicons (IncFIA, IncFIB, and IncFII). Four variants of AMEs were detected in the U7 isolate [*aac(3′)-IIa*, *aac(6′)-Ib-cr*, *aph(3″)-Ib*, *aph(6)-Id*)]. The *aac(6′)-Ib-cr* determinant has been found as the most prevalent gene in *E. coli* from clinical samples and is commonly associated with *bla_CTX-M-15_* and *bla_OXA-1_* [66]–[68]. This association was found for UTI-U7, which exhibited resistance to gentamicin, ciprofloxacin, and norfloxacin. Moreover, mutations in *gyrA* and *parC* were recognized in UTI-U7; these substitutions correlate with augmented quinolone resistance in clinical isolates from UTIs [69],[70]. Twenty-one ExPEC-associated genes were identified. Those involved in adherence (*iha*, *hra*, *papA_F19/F20*, *papC*, and *yfcV)* and iron uptake (*chuA*, *fyuA*, *irp2*, *iucC*, and *sitA*) accounted for most of the virulence genes. These virulence factors are essential for the survival of UPEC in an environment different from the gut, as well as for its pathogenic mechanism, being involved in adherence, nutriment acquisition, colonization, invasion, replication, and biofilm formation [10],[71]. The prevalence of virulence genes is variable, but most studies describe the adherence genes as the most prevalent in uropathogenic strains [2],[72]. Several studies of clinical *E. coli* isolates from Mexico have reported *fimH* and *papC* as prevalent genes [73]–[74]; however, only *papC* was identified in the UTI-U7 isolate. The toxin-associated genes (*cnf1*, *hlyA*, *sat*, and *usp*) detected in the analysis are frequent in ExPEC strains and might play a potential role in complicated infections [10],[75]. After an extensive genomic analysis and the noticeable resistance and virulence potential of UTI-U7, coupled with the need for new treatment options to combat these strains, MEs of different *Echeveria* species (*E. craigiana, E. kimnachii*, and *E. subrigida)* were evaluated as potential antibacterial treatments. All extracts showed antibacterial activity against the UTI-U7 isolate, highlighting the effect of *the E. subrigida* extract (MIC 5 mg/mL) versus the other extracts (MIC 25 and 50 mg/mL for *E. craigiana* and *E. kimnachii*, respectively). In accordance with the latter, previous reports have demonstrated good antibacterial effects of *E. subrigida* against Gram-positive bacteria, but poor effects against Gram-negative organisms such as *E. coli*, when evaluated up to 1 mg/mL of extract. Such variability is associated with differences in the bacterial cell walls and extract constituents, of which has been previously determined that *E. subrigida* contains flavonoids, coumarins, and tannins with potential antibacterial activity [18],[76]. With these in mind and literature evidence, we decided to evaluate higher concentrations against the Gram-negative UTI-U7. These same extracts were proven in diarrheagenic *E. coli* reference strains, with a significant reduction in bacterial growth and decreased oxygen consumption, which is a similar effect to that exerted by bacteriostatic antibiotics. Additionally, *Echeveria subrigida* showed the best effects on these pathogenic strains [19]. Once the MIC of the extracts was determined, adherence assays were performed to determine whether the *Echeveria subrigida* extract could influence the initial step of UPEC infection. Adherence is essential for the establishment of a UTI and a triggering event for the subsequent steps of the pathogenic mechanism [77]. Currently, anti-adherence therapies offer an alternative to bacterial infections by blocking the interaction of bacteria with epithelial cells without killing them, thus eliminating the bacteria. Plant polyphenols have been shown to block bacterial adhesion [78]. It is important to note that these constituents and a bacteriostatic effect have been found in *Echeveria* extracts [18],[19]. No significant differences were found between treated and untreated bacteria (1.29 × 10^7^ vs 1.18 × 10^7^ CFU, respectively). To the best of our knowledge, this is the first study to investigate the antibacterial antiadherence properties of *Echeveria* plant extracts. Most of the studies evaluated the antibacterial properties of members of the Crassulaceae family and only determined the MIC; however, none of these studies evaluated whether the extracts had any effect in bacterial adherence [79]. Although effects on adherence were not observed in the present study, further analyses are needed to evaluate other possible mechanisms by which the *Echeveria* extracts could participate against bacterial infection.

## 5. Conclusions

UTI-U7 is an MDR isolate with a resistant determinant to aminoglycosides, β-lactams, fluoroquinolones/quinolones, amphenicols, and tetracyclines, which correlates with its resistance profile. In addition, the identified virulence genes corroborated the pathogenic potential of this circulating isolate. The results of this study contribute to the relevance of the molecular epidemiologic surveillance of these pathogenic strains, especially in countries where these strategies are scarce, with the aim of decreasing their spread. Additionally, it is important to continue searching for alternative treatments (e.g., plant extracts) for these infectious diseases to combat antimicrobial resistance. In this context, further studies must determine the potential of the *E. subrigida* extract as an antibacterial to be used to treat UTIs.


